# Metagenomic analysis reveals diverse microbial community and potential functional roles in Baner rivulet, India

**DOI:** 10.1186/s43141-023-00601-x

**Published:** 2023-11-28

**Authors:** Bhavna Brar, Ravi Kumar, Dixit Sharma, Amit Kumar Sharma, Kushal Thakur, Danish Mahajan, Rakesh Kumar

**Affiliations:** 1 Department of Animal Sciences, School of Life Sciences, Central University of Himachal Pradesh, Kangra, Himachal Pradesh India; 2 Department of Microbiology, Dr. Rajendra Prasad Government Medical College & Hospital, Tanda, Kangra, Himachal Pradesh India

**Keywords:** Microbial diversity, Metagenomics, River, e-DNA, Beas

## Abstract

**Background:**

The health index of any population is directly correlated with the water quality, which in turn depends upon physicochemical characteristics and the microbiome of that aquatic source. For maintaining the water quality, knowledge of microbial diversity is a must. The present investigation attempts to evaluate the microflora of Baner. Metagenomics has been proven to be the technique for examining the genetic diversity of unculturable microbiota without using traditional culturing techniques. The microbial profile of Baner is analyzed using metagenomics for the first time to the best of our knowledge.

**Results:**

To explore the microbial diversity of Baner, metagenomics analysis from 3 different sites was done. Data analysis identified 29 phyla, 62 classes, 131 orders, 268 families, and 741 genera. *Proteobacteria* was found to be the most abundant phylum in all the sampling sites, with the highest abundance at S_3_ sampling site (94%). *Bacteroidetes* phylum was found to be second abundant in S_1_ and S_2_ site, whereas *Actinobacteria* was second dominant in sampling site S_3_. *Enterobacteriaceae* family was dominant in site S1, whereas *Comamonadaceae* and *Pseudomonadaceae* was abundant in sites S_2_ and S_3_ respectively. The Baner possesses an abundant bacterial profile that holds great promise for developing bioremediation tactics against a variety of harmful substances.

**Conclusion:**

Baner river’s metagenomic analysis offers the first insight into the microbial profile of this hilly stream. *Proteobacteria* was found to be the most abundant phylum in all the sampling sites indicating anthropogenic interference and sewage contamination. The highest abundance of *proteobacteria* at S_3_ reveals it to be the most polluted site, as it is the last sampling site downstream of the area under investigation, and falls after crossing the main city, so more human intervention and pollution were observed. Despite some pathogens, a rich profile of bacteria involved in bioremediation, xenobiotic degradation, and beneficial fish probiotics was observed, reflecting their potential applications for improving water quality and establishing a healthy aquaculture and fishery section.

## Background

Water covers around 75% of the earth’s crust, but nearly 3% of that water is fresh, and 99% of that 3% is trapped in glaciers, polar ice caps, or reservoirs. So, only a trace of the whole water of this earth is available as fresh water. The hydrosphere is an essential part of a sustainable environment, life originated in water, and even the origin and development of human civilization are closely related to the river. So, preserving valuable water resources and aquatic ecosystems is of utmost importance [53]. The quality of freshwater resources has significantly declined in recent years due to population growth, rapid urbanization, industrial effluents, using fertilizers and pesticides for agricultural purposes, and waste disposal. Sustainable water management of a river is quite essential to maintain stability. The effects of water pollution incidents on water quality safety and local inhabitants’ quality of life are currently receiving global attention [20, 25, 89]. In India, several major rivers have been found to exhibit excessive levels of pollution that have adversely affected the ecosystem and human population [41, 50, 88, 34, 14]. Community structure and function dynamics across contaminated rivers are essential for understanding and assessing human activities’ impacts on water ecosystems [63]. In the present scenario, there is an increasing need for regular monitoring and assessment of aquatic ecosystems so that further steps may be taken to preserve the sustainability of these resources. Comprehensive monitoring involves various techniques like physiochemical, hydrological, and biological approaches giving the exact status of the aquatic ecosystem. The latest trend in biomonitoring the ecology of aquatic systems is metagenomics through environmental DNA (e-DNA). Free DNA molecules, or “eDNA,” are those nucleic acids that exist outside of organisms, in the surrounding environment like soil, water, and snow [12]. They may be shed through skin, saliva, gametes, excreta, or corpus remains. Whole-genome shotgun sequencing (WGS based on next-generation sequencing (NGS is used to create a metagenome from eDNA which is taken straight from the environment, revealing complete genetic information of all the organisms present in that particular environment in a single stretch [87, 64]. In situations where collecting whole organisms is difficult or not feasible, or in case of cryptic, endangered, hidden, invasive species, unculturable bacterial species, the fast-expanding study of eDNA has proved a boon to identifying species and conducting genetic analyses for conserving, managing, and research through metagenomics. Microbes are significant organisms in freshwater habitats that can play a role in a variety of ecological events. Bacteria and fungi play a significant role in the conversion of biological and non-biological materials by participating in numerous biogeochemical cycles such as the nitrogen, carbon, sulfur, and phosphorus cycles, which are responsible for the ecosystem’s health and balance. Thorough understanding of the microbial variety and their functionalities found in freshwater bodies is critical for their long-term management of aquatic ecosystems [33, 76].

Bacteria are the most prevalent creatures on the planet; their composition as well their richness highly affects the ecosystem functions and stability whether they are present in host-associated colonies, soil, grassland, or seas [100, 8, 67, 91, 96, 104]. Aquatic microbiomes play crucial roles in nutrient recycling and aquatic ecology functioning. Various estimations of cell density, volume, and carbon reveal that prokaryotes of aquatic habitats are cosmopolitan, and amidst the wide variety of cell densities that have been observed till now, the average values of bacterial composition for several aquatic habitats are remarkably similar [100, 76]. The use of river water directly for drinking poses severe risks as anthropogenic activities generate environmental pollution [9]. Understanding the composition of the bacterial populations in a freshwater habitat is one of the essential steps in ensuring the health of that particular ecosystem [32]. Moreover, compared to marine pathogenic bacteria affecting human health, freshwater bacteria are less explored [30]. Kangra Valley’s residents rely heavily on the portability of Baner rivulet water [14]. The emergence of next-generation sequencing technologies and computational biology has open up the new horizons for mining the genome, transcriptome, and proteome sequence data. The bioinformatics approach for characterization and classification of genes and proteins is time-consuming, cost-effective, and less laborious and makes it possible to mine huge biological dataset [82, 84, 85]. The present investigation was an attempt made to evaluate the microflora of Baner rivulet using the metagenomics technique. The complete microbial profile of Baner is analyzed for the first time to the best of our knowledge.

## Methods

### Study area

**Fig. 1:**
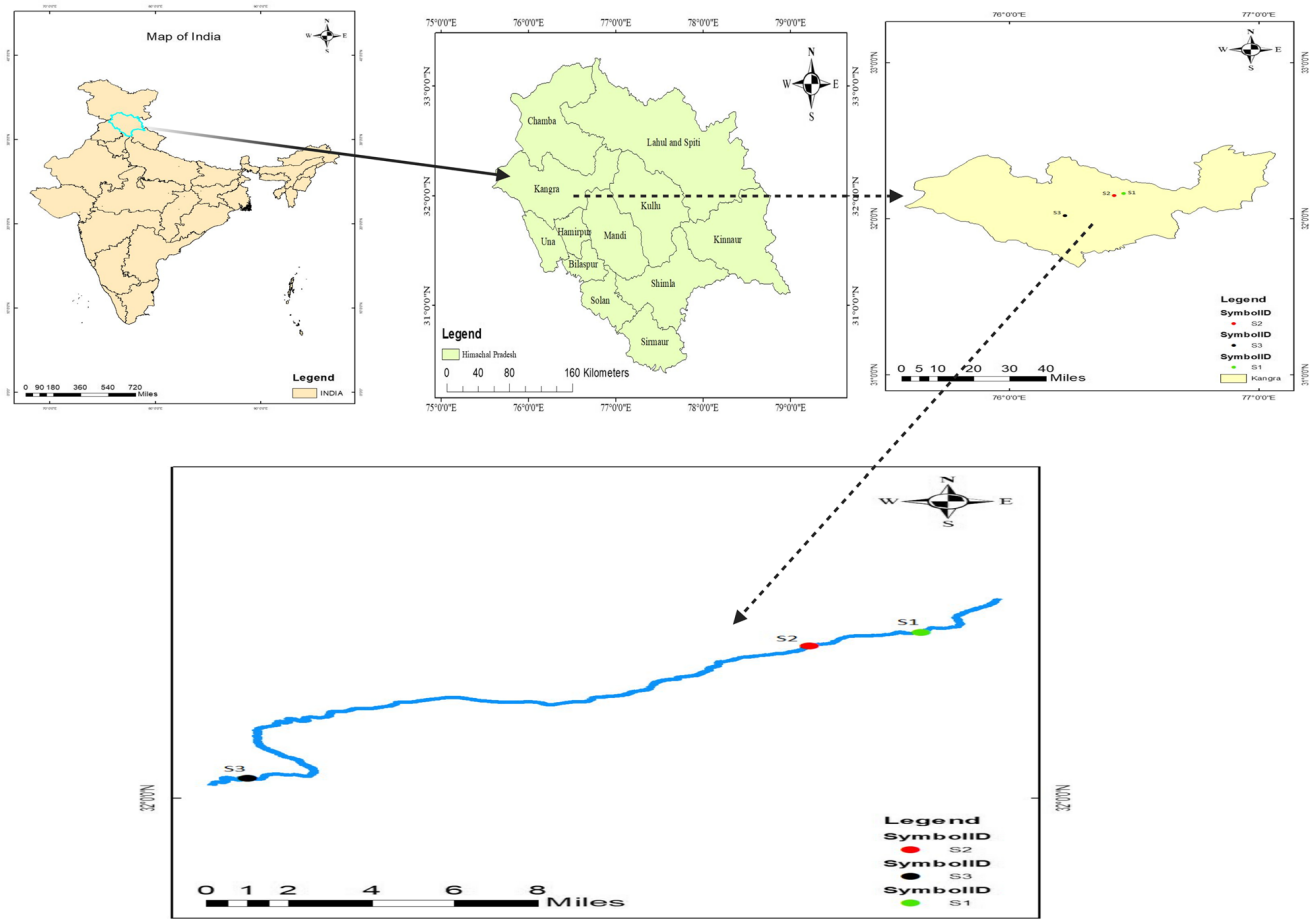
Location map showing three sampling sites of Baner

Baner rivulet in Kangra, Himachal Pradesh, the site of the current investigation, is an essential perennial tributary of river Beas, which is one of the major rivers of the Kangra district. The Baner rivulet is one of the main sources of drinking water for the people residing in district Kangra. Baner, with an area of 668 km^2^, originates in the southern slopes of snow-capped Dhouladhar Mountains near Aadi Himani Chamunda Temple, Palampur, Himachal Pradesh, India (Table 1; Fig. 1). It drains the central part of Kangra district and fans in a south-westward direction before merging with Pong Dam close to Mahora Village in district Kangra [84, 85]. The area of the investigation for Baner rivulet was divided into three sites. The sampling sites which were selected downstream were S_1_ (Jia), S_2_ (Chamunda Shaktipeeth), and S_3_ in Ranital near the confluence of Bathu rivulet and Baner.

**Table 1:** Sampling sites with geo-coordinates

Sampling site	Name	Latitude	Longitude
S_1_	Jia	32° 9′ 46.49076	76° 27′ 34.236
S_2_	Chamunda	32° 8′ 46.44852	76° 24′ 52.35156
S_3_	Bathu (Ranital)	32° 01′ 14.00″	76° 14′ 35.00″

### Sample collection, analysis and sequencing

 The 250 ml of surface water sample was collected in sterile polyethylene bottles from all three sampling sites, i.e., S_1_ (Jia), S_2_ (Chamunda Shaktipeeth), and S_3_ (Ranital). The sample was carried to the laboratory at 4 °C and stored at – 20 °C till further analysis. The debris and coarse particles were removed by filtering the sample through 1.2 μm pore size membrane. Furthermore, the sample was passed by 0.2 μm pore membrane for collecting prokaryotes cell in sample. Metagenomic DNA was isolated from the samples from each site utilizing the DNeasy PowerWater kit (QIAGEN) as per the manufacturer’s protocol. All laboratory procedures were performed following strict protocols. Enzymatic DNA fragmentation was done using KAPA fragkit to produce small double-stranded DNA segments. The library preparation was started with 200 ng g-DNA. KAPA Hyper Prep kit was used for Library Preparation from these dsDNA fragments. End repair and A tailing, i.e., the addition of A at 3′ end to the dsDNA, was followed by adapter ligation. The adapters’ sequences complement the dual-barcoded libraries in a flow cell for sequencing, enable PCR amplification of adapter-ligated fragments, and bind the conventional Illumina sequencing primers. The library amplification of adapter-ligated libraries was done using KAPA HiFi Hot Start Ready Mix and KAPA Library Quantification Kit. Sequencing was done by Novaseq 6000 Illumina to generate raw data in the form of fastq reads.

### Bioinformatics analysis of Baner metagenome

#### Data generation and quality check

The raw fastq data generated by sequencing were checked for noise and processed by computational tools. The data was pre-processed. The reads were trimmed two from the front and three from the tail and filtered on the parameter of < Q20. The data quality was improved after trimming and filtering, and high-quality reads were used for downstream analysis.

#### De-novo whole gene assembly

The metaSPAdes-St. Peterburg genome assembler (Megahit genome assembler) was used for the de novo assembly of microbial genomes. MetaSPAdes tool combines the latest algorithmic ideas with already proven solutions from the SPAdes toolkit to address different challenges of metagenomic assembly [65]. For the complete assessment of organisms in the sample, CCmetagen 1.3 tool was used to analyze the diversity from high-throughput sequencing of DNA [59]. After the de novo assembly of sequence data, contigs in FASTA format were obtained for every sample. Following assembly, the contigs were uploaded to Prodigal to anticipate open reading frames (ORFs) [40]. The coding regions have been recognized and separated from noncoding DNA using the gene discovery tool Prodigal.

#### Taxonomic profiling and annotations of scaffolds

Taxonomic profiling was carried out on all the metagenomics samples using National Centre for Biotechnology Information (NCBI) taxonomy datasets. Each sequencing read was assigned to a taxon in the NCBI taxonomy compared to a reference database with nucleotide sequences. It uses the set of available complete non-redundant Nucleotide databases (nr Database) that includes fungi and microbial eukaryotes genomes. The similarity search of reads was performed at NCBI using BLAST with default parameters [61]. Furthermore, the taxonomy file is used for Krona plot generation using the Krona tool [66]. Functional annotations of all the contigs were performed using SEED classification. Each contig’s function was assigned using the MGA software. The protein functions of each contig having maximum alignment score from MGA results selected for the functional assignment. Pathway analysis was performed using the kofam software at KEGG Database [44].

## Results

**Fig. 2:**
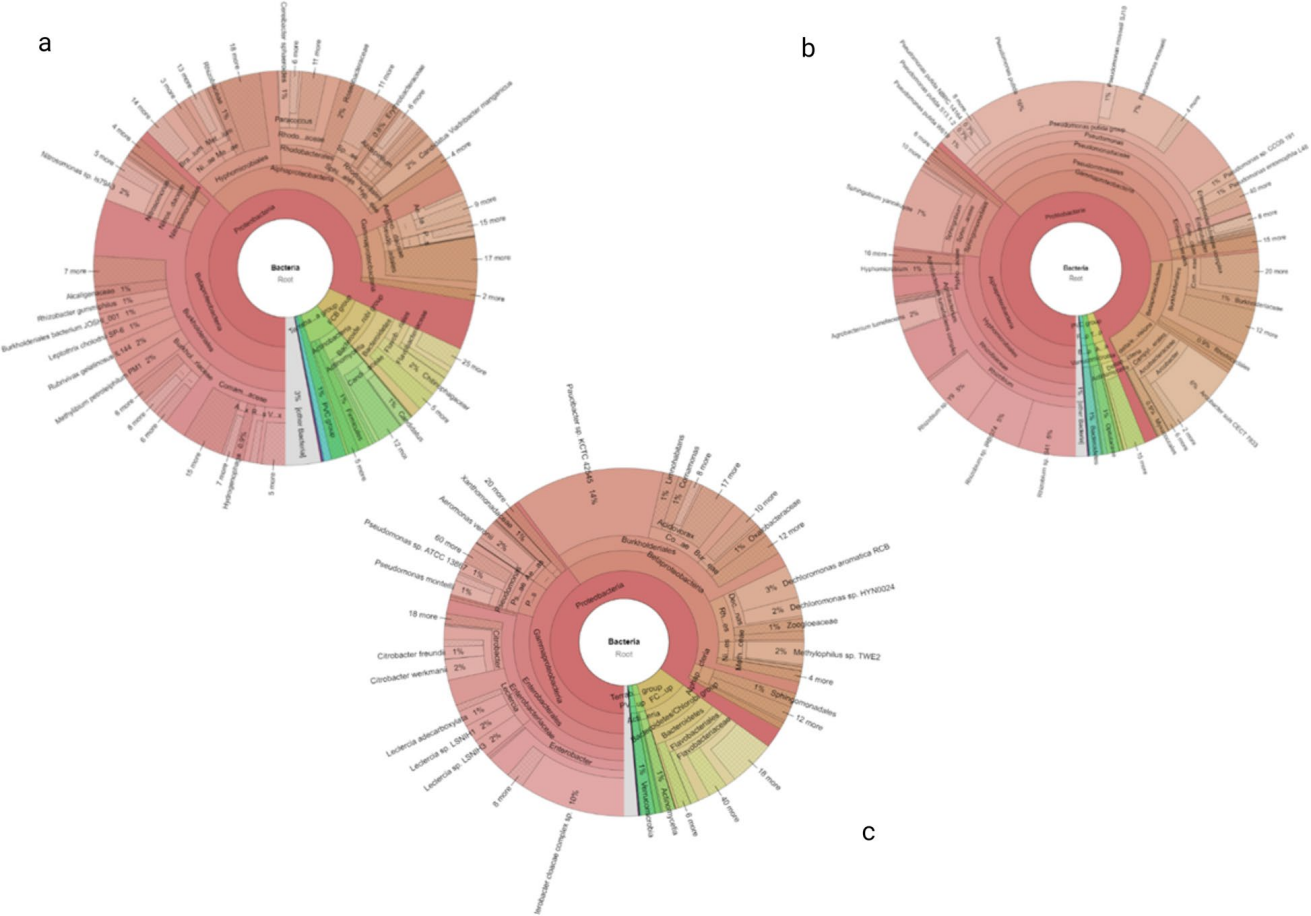
Krona graph showing taxonomy at species level at sampling sites. **a** S_1_. **b** S_2_. **c** S_1_

Metagenomics analysis revealed the identification of 29 phyla, 62 classes, 131orders, 268 families, and 741 genera. Krona graphs (Fig. 2) have been used to show the entire taxonomy, from kingdom up to species level [66]. The detailed description of the data has been given as a comparative account at different taxonomical levels (Tables 2, 3, 4, 5, and 6). The microbiome of Baner showed phylum-wise dominance of *Proteobacteria* (23,655, 85%), followed by *Bacteroidetes* (2815, 10%), *Actinobacteria* (367, 1%), and *Verrucomicrobia* (307, 1%), at S_1_. In contrast, at S_2_, it was *Proteobacteria* (3270, 82%), *Bacteroidetes* (272, 7%), *Actinobacteria* (165, 4%), and *Firmicutes* (40, 1%), which is the same pattern as that of S_1_, while at S_3_, phylum level analysis revealed a dominance of *Proteobacteria* (11,517, 94%), *Actinobacteria* (245, 2%), *Verrucomicrobia* (188, 1.9%), and *Bacteroidetes* (128, 1%) (Table 2).

**Table 2:** Taxonomy abundance at phylum level (top 4)

S1	S_2_	S_3_
*Proteobacteria* (85%)	*Proteobacteria* (82%)	*Proteobacteria (*94%)
*Bacteroidetes* (10%)	*Bacteroidetes* (7%)	*Actinobacteria* (2%)
*Actinobacteria* (1%)	*Actinobacteria* (4%)	*Verrucomicrobia* (1.9%)
*Verrucomicrobia* (1%)	*Firmicutes* (1%),	*Bacteroidetes* (1%)

**Table 3:** Taxonomy abundance at class level (top 4)

S1	S2	S3
*Gammaproteobacteria* (40%)	*Betaproteobacteria* (37%)	*Alphaproteobacteria* (37%)
*Betaproteobacteria* (40%)	*Alphaproteobacteria* (31%)	*Gammaproteobacteria* (36%)
*Alphaproteobacteria* (3%)	*Gammaproteobacteria* (8%)	*Betaproteobacteria* (11%)
*Actinomycetia* (1%)	*Actinomycetia* (4%)	*Actinomycetia* (2%)

**Table 4:** Taxonomy abundance at order level (top 4)

S1	S2	S3
*Enterobacterales* (29%)	*Burkholderiales* (31%)	*Pseudomonadales* (33%)
*Burkholderiales* (28%)	*Hyphomicrobiales* (12%)	*Hyphomicrobiales* (27%)
*Rhodocyclales* (7%)	*Rhodobacterales* (7%)	*Burkholderiales* (9%)
*Pseudomonadales* (5%)	*Nitrosomonadales* (4%)	*Sphingomonadales* (9%)

**Table 5:** Taxonomy abundance at family level (top 4)

S1	S2	S3
*Enterobacteriaceae* (29%)	*Comamonadaceae* (11%)	*Pseudomonadaceae* (33%)
*Flavobacteriaceae* (9%)	*Rhodobacteraceae* (5%)	*Rhizobiaceae* (24%)
*Comamonadaceae (8%)*	*Nitrobacteraceae (4%)*	*Sphingomonadaceae (8%)*
*Pseudomonadaceae* (5%)	*Nitrosomonadaceae* (4%) *Burkholderiaceae* (4%)	*Acrobacteriaceae* (7%)

**Table 6:** Taxonomy abundance at genus level (top 4)

S1	S2	S3
*Enterobacter* (14%)	*Nitrosomonas* (4%)	*Pseudomonas (33*%*)*
*Paucibacter* (14%)	*Bradyrhizobium* (3%)	*Rhizobium (20*%*)*
*Leclercia* (8%)	*Aeromonas* (3%)	*Sphingobium (8*%*)*
*Citrobacter* (5%) *Pseudomonas* (5%)	*Variovorax* (2%)	*Pseudoarcobacter* 7%)

**Fig. 3:**
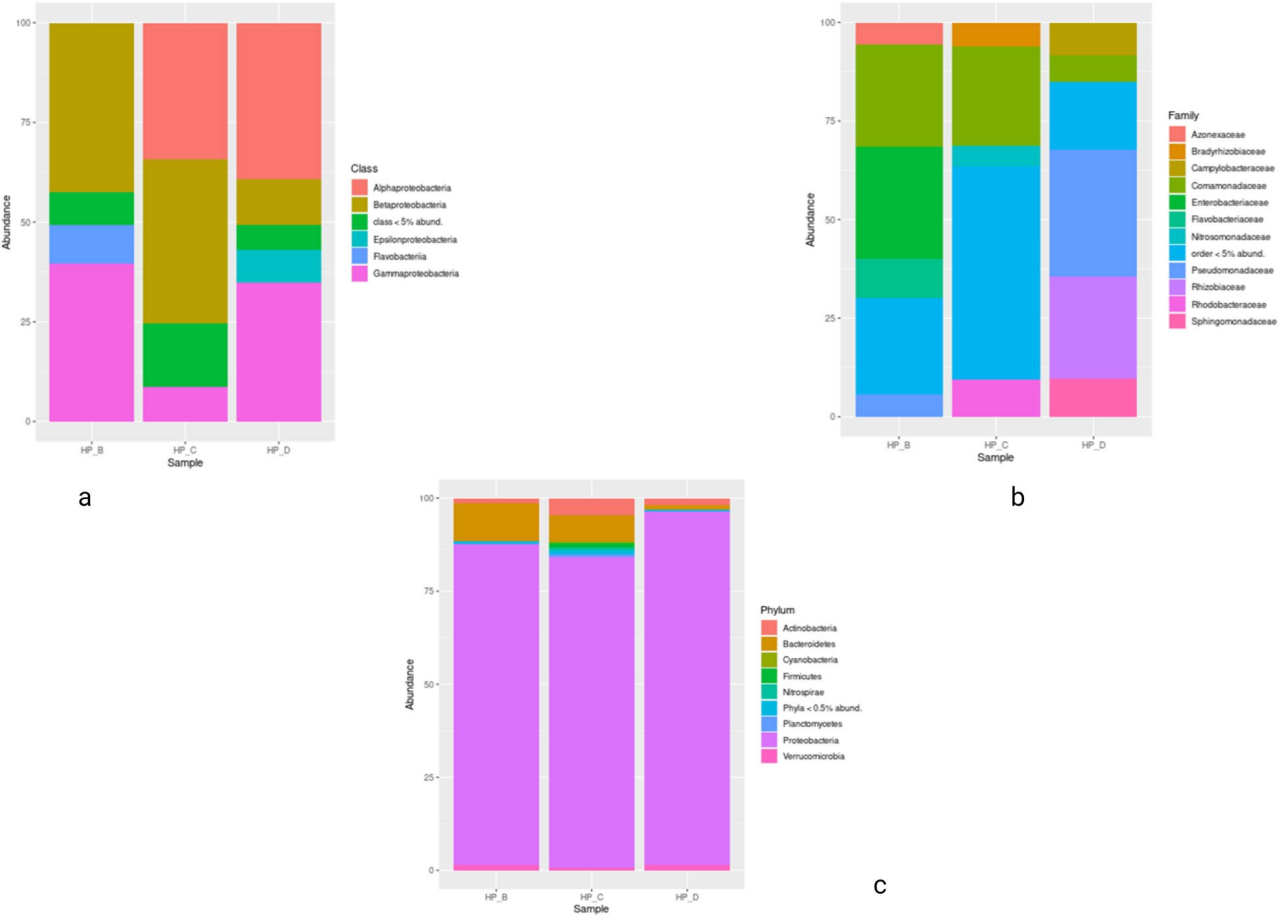
Comparative graphs showing relative taxonomic abundances at different taxonomic hierarchy levels at S_1_ (HP_B), S_2_ (HP_C), and S_3_ HP_D). **a** Class. **b** Family. **c** Phylum

At the class level, the order of dominance in decreasing order at S_1_ is *Gammaproteobacteria* (11,099, 40%), *Betaproteobacteria* (11,049, 40%), *Alphaproteobacteria* (797, 3%), and *Actinomycetia* (316, 1%). At S_2_, the order was as *Betaproteobacteria* (1487, 37%), *Alphaproteobacteria* (1257, 31%), *Gammaproteobacteria* (328, 8%), and *Actinomycetia* (143, 4%). At S_3_, it was *Alphaproteobacteria* (4520, 37%), *Gammaproteobacteria* (4508, 36%), *Betaproteobacteria* (1323, 11%), and *Actinomycetia* (237, 2%) (Table 3; Fig. 3a).

The order level analysis at S_1_ resulted in the dominance of *Enterobacterales* (8171, 29%), *Burkholderiales* (7716, 28%), *Rhodocyclales* (1988, 7%), *Pseudomonadales* (1470, 5%), and *Nitrosomonadales* (756, 2%). At S_2_, the dominance order was *Burkholderiales* (1227, 31%), *Hyphomicrobiales* (487, 12%), *Rhodobacterales* (298, 7%) *Nitrosomonadales* (180, 4%), and *Sphingomonadales* (146, 3%). At S3, *Pseudomonadales* (4104, 33%), *Hyphomicrobiales* (3301, 27%), *Burkholderiales*(1127, 9%), and *Sphingomonadales* (1063, 9%) are shown in Table 4.

At the family level, the dominance at S_1_ was as follows (Table 5): *Enterobacteriaceae* (8023, 29%), *Flavobacteriaceae* (2431, 9%), *Comamonadaceae* (2251, 8%), and *Pseudomonadaceae* (1463, 5%). At S_2_, it was *Comamonadaceae* (430, 11%), *Rhodobacteraceae* (213, 5%), *Nitrobacteraceae* (180, 4%), *Burkholderiaceae* (163, 4%), and *Nitrosomonadaceae* (163, 4%). At S_3_, it was *Pseudomonadaceae* (4097, 33%), *Rhizobiaceae* (2996, 24%), *Sphingomonadaceae* (1022, 8%), and *Acrobacteriaceae* (882, 7%), (Fig. 3b).

The dominance pattern at the genus level included (Table 6) *Enterobacter* (3819, 14%), *Paucibacter* (3756, 14%), *Leclercia* (2144, 8%), *Citrobacter* (1448, 5%), and *Pseudomonas* (1443, 5%), at S_1_, while at S_2_, it was *Nitrosomonas* (152, 4%), *Bradyrhizobium* (117, 3%), *Aeromonas* (104, 3%), *Variovorax* (95, 2%), *Rubrivivax* (76, 1%), and *Methylibium* (76, 1%), respectively. At S_3_, it was *Pseudomonas* (4092, 33%), *Rhizobium* (2401, 20%), *Sphingobium* (918, 8%), and *Pseudoarcobacter* (780, 7%).

**Fig. 4:**
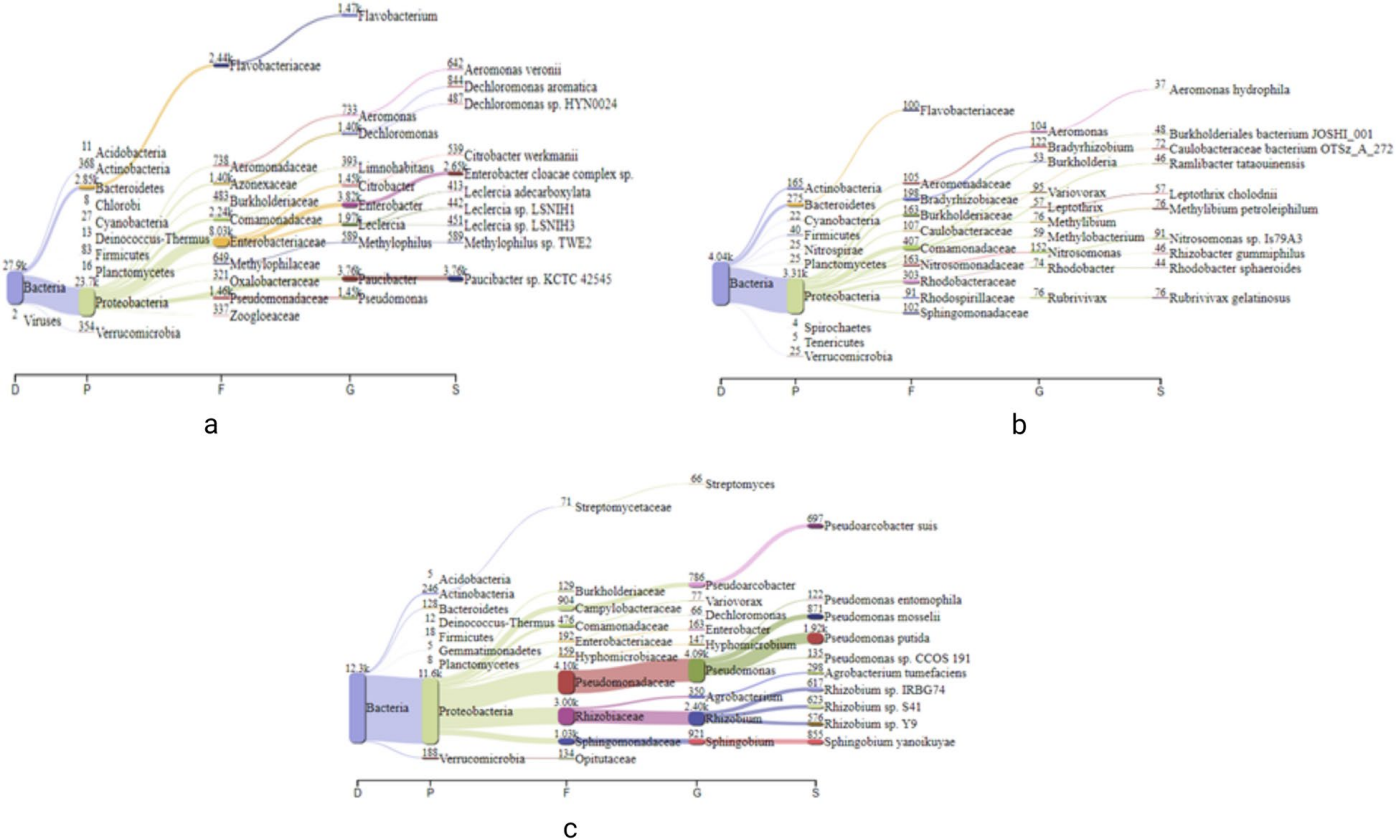
Sankey diagrams showings composition of microbial diversity at division, phylum, family, genus, and species levels. **a** S_1_. **b** S_2_. **c** S_3_

At the species level, metagenomic analysis revealed the order of dominance at S_1_ (Table 7, Fig. 4a) as *Paucibacter* sp. *KCTC42545* (3756, 14%), *Enterobacter cloacae* complex sp. (2648, 10%), *Dechloromonas aromatica* (844, 3%), and *Methylophilus* sp. *TWE2* (589, 2%). At S_2_, as shown in Fig. 4b, it was as *Nitrosomonas* species (91, 2%), *Methylibium petroleiphilum PM1* (76, 2%), *Rubrivivax gelatinous IL144* (76, 2%), and *Caulobacteraceae bacterium* (37, 0.9%), and at *S* *3*, presented in Fig. 4c, the order was *Pseudomonas putida* (1918, 16%), *Pseudomonas mosselii* (871, 7%), *Sphingobium yanoikuyae* (855, 7%), and *Pseudoarcobacter suis* (697, 6%).

**Table 7:** Taxonomy abundance at species level (top 4)

S1	S2	S3
*Paucibacter* sp. *KCTC42545*(14%)	*Nitrosomonas* sp. (2%)	*Pseudomonas putida* (16%)
*Enterobacter cloacae complex* sp. (10%)	*Methylibium petroleiphilum* (2%*)*	*Pseudomonas mosselii* (7%)
*Dechloromonas aromatic* (3%)	*Rubrivivax gelatinous* (2%)	*Sphingobium yanoikuyae* (7%)
*Methylophilus* sp. *TWE2* (2%)	*Caulobacteraceae bacterium* (0.9%)	*Pseudoarcobacter suis* (6%)

Taxonomic hierarchy along with relative percentage of different taxa from sampling sites

## Discussion

In general, freshwater habitats have microbial taxa that are different from those found in marine and terrestrial ecosystems. Various estimations of cell density, volume, and carbon reveal that prokaryotes of freshwater habitats are cosmopolitan, and amidst the wide variety of cell densities that have been observed till now, the average values of bacterial composition for several aquatic habitats are remarkably similar [100]. All freshwater forms are more likely to include *Betaproteobacteria*, *Actinobacteria*, *Bacteroidetes*, *Verrucomicrobia*, and *Alphaproteobacteria* [30, 51]. Similar findings were observed during the present study. *Proteobacteria* were found to be the most prevalent and dominating phylum in all three sampling sites (Fig. 3c), indicating anthropogenic interference and municipal disposal [102]. They were most abundant at S_3_, revealing it to be the most polluted site, as it is the last sampling site downstream, of the area under investigation, which shows more human intervention and pollution, as this area falls after crossing the main city. A similar pattern was observed by the researchers in earlier studies [42, 56]. *Proteobacteria* are mostly fast-growing copiotrophs that can thrive in environments with abundant nutrients, and they are thought to be crucial for nitrogen cycling, linking iron-carbon biogeochemistry, carbon sequestration, nutrient flux, and other biochemical phenomena [52]. The Baner rivulet’s metagenomic analysis initially showed a diverse microbial makeup revealing a complex microbial composition of both helpful and unfriendly species, detailed in the following section.

### Beneficial microbiota of Baner

#### Bio-remedial bacterial profile

Several prokaryotic and eukaryotic species have evolved defense systems against toxic metals, making them benign. With the help of their genes, microbes typically biosorb metals and sequester these in their cell walls [17, 79]. They adopt various mechanisms to respond to heavy metal stress, such as exclusion, compartmentalization, complex formation, and the creation of binding proteins like metallothioneins (MTs) [80–83]. During the present investigation, some of bio-remedial species were found in Baner that can be utilized to remove heavy metals, harmful chemicals, pesticides, etc.

Few strains of cyanobacterium *Synechococcus* which produce MT have been discovered. Compared to mammalian MTs, prokaryotic MT has fewer cysteine residues [57]. *Synechococcus* has been found at all three selected sites of Baner and can reduce copper, cadmium, and zinc from the environment [103].

*Leptothrix discophora*, gram-negative bacteria, showed the capability of oxidizing Mn(II) [13]. It extracts manganese from the environment, synthesizes Mn(II)-oxidizing proteins as a component of an extracellular sheath matter for the purpose of growth; defense against predation, UV light, or viral attacks; and probably also for immobilizing the toxic metal [2, 92]. The bacteria have the ability to produce two different extracellular macromolecules that catalyze the oxidation of Fe(II) and Mn(II) which makes it useful for bioremediation [21, 95]. The metal-encrusted surfaces of Mn(II)-oxidizing bacteria provide biogenic Mn(II) oxidation which can be used for the removal of Mn(II), Fe(II), and As(III) from potable groundwater. The role of *Leptothrix* sp. and *Gallionella* sp. in treating the groundwater for Mn(II) and Fe(II) removal has been suggested by Katsoyiannis and Zouboulis  [46, 47]. Raw water is treated biologically by Mn(II)-oxidizing bacteria, including *Leptothrix cholodnii*, in biological filters extensively [46, 47]. Both *Gallionella* and *Leptothrix* have been found at all three sampling sites (S_1_, S_2_, and S_3_).

*Acidovorax sp*. is a mesophilic gram-negative bacterium decomposing nitroarene compounds to use 2-nitrotoluene (2NT) as the only source of carbon and energy [58]. Nitroarene compounds are a class of poisonous chemicals predominantly man-made and known to contaminate soil and groundwater, including chemical manufacturing units and explosive factories, grenades, and detonation sites [36]. *Pseudomonas putida*, the most abundant species at S_3_, was genetically engineered, increasing its inherent cadmium binding capacity threefold. Similarly, the metal binding capacity of *Burkholderia*, *Alcaligenes*, and *Ralstonia* can also be enhanced so that further they may be utilized to eliminate heavy metal pollution from sewage and industrially polluted water bodies [81, 94]. All these species were abundant in Baner rivulet.

*Arthrobacter* is known to possess growth-promoting activity in plants; these bacteria also possess genes for uptaking heavy metals, to decompose complex organic and inorganic compounds. Their respective genes for diverse metal degradation can be utilized for synthesizing transgene, which can then be utilized to create artificial bacteria that can degrade various xenobiotics [71]. The role of *Arthrobacter* in bioremediation has been proved multiple times through the removal of pesticides and herbicides, pollutants like 4 chlorophenol, pentachloronitrobenzene cypermethrin, cyhalothrin, dichlorobiphenyls, trichloroethylene, p-nitrophenol, and atrazine or removing heavy metal like chromium and iron [11, 43, 62, 69, 98, 101].

Rare earth metals cerium and neodymium are bioaccumulated by *Bacillus cereus* [17]. The involvement of *Pseudomonas* in iron and uranium removal has been proved earlier [39]. *Methylibium petroleiphilum* plays an essential role in bioremediation by eliminating MTBE ( methyl *tert*-butyl ether), a component of gasoline [35].

*Paucibacter* is good at remediation of Microcystis algal blooms and microcystin; microcystin is a carcinogen and hepatotoxin for humans and causes mortalities in fish and livestock [54, 70]. *Leptothrix* and *Dechloromonas* are sulfur oxidation bacteria converting hydrogen sulfide to sulfur element or sulfate and removing toxic compounds with foul-smell like H_2_S [19, 106]. *Dechloromonas aromatica* plays important role in the biodegradation of benzene [73]. *Sphingobium* sp. degrades phenanthrene (component of plastics), pesticides, explosives, and drugs. Aromatic molecules, glycan polymers, metal ions, xenobiotics, and resistive substances are all catabolized by the *Variovorax paradoxus* [74]. *Acinetobacter* sp., *Klebsiella* sp., and *Elizabethkingia* sp. efficiently biodegrade ciprofloxacin and levofloxacin [77]. *Rhizobacter gummiphilus*, found at all three sites, participates in the degradation of rubber, a component of tyres and surgical gloves [45]. *Rhodobacter sphaeroides* have an excellent potential for heavy metals bioremediation [5, 15]. *Stenotrophomonas maltophilia* has been found at both S_2_ and S_3_ which has been proven to transform As(V) to As(III) and nontoxic arsenobetaine in the fish gut [90].

#### Potential probiotics for fish

During the present study, several probiotic bacteria were found in Baner which are helpful for humans as well as aquatic organisms. Probiotics are microorganisms or derivatives from them that provide good health and growth of the host that are used in aquaculture to manage the disease, complement immunity, and in some circumstances even substitute the use of antibacterial agents like antibiotics. Fish are benefited in many ways through these probiotic bacteria, including growth promotion, pathogen colonization inhibition, improved digestion, water quality, stress tolerance, and fertility improvement [23]. The findings of the studies on fish probiotics were reported earlier [16] which showed that the bacteria present in the aquatic systems impacted the gut microbial diversity and vice versa. Species that can survive and proliferate in the digestive system often appear to be those from the surroundings or the food.

The probiotics can offer a promising approach for controlling the bacterial, fungal and parasitic infections in fishes [93]. The role of *Enterococcus faecium* as a probiotic for improving the health of *Labeo rohita* has been proved previously [31]. The *Bacillus* sp. has also been recognized a good fish probiotic involved in overall development [37]. *Bacillus subtilis* and *B*. *circulans* enhanced the fish growth and eliminated the anti-nutritional factor mimosine [6, 7]. *Lactobacillus* sp. also shows good probiotic potential, inhibiting activities against harmful fish pathogen *Aeromonas hydrophila* and boosting fish health [4, 27, 75]. *Micrococcus* and *Pseudomonas* are good as probiotics for enhancing fish growth [1]. The probiotic activity of *Roseobacter* against *Vibrio anguillarum* infection has been proved efficient earlier [22, 38, 68]. *Rubrivivax gelatinosus* is a probiotic as a bacterium, and its biomass and by-products boost the immune system of fish to combat pathogens and enhance growth [28, 29]. Sharifuzzaman and Austin [78] observed that the cellular components of *Rhodococcus* improved rainbow trout resistance against *Vibrio anguillarum*. The probiotic activity of *Alteromonas* sp., *Phaeobacter gallaeciensis*, and *Pseudomonas damselae* against *Vibrio splendidus* and *V*. *coralliilyticus* has been reported [48, 49]. *Shewanella putrefaciens* and *Shewanella baltica* were examined for their probiotic potential in fishes which heightened immune response and increased resistance to *Photobacterium damselae* [24]. All abovementioned probiotic bacteria have been found in the microbial profile of Baner.

#### Pathogens

Several disease-causing pathogens have been found during the metagenomics study of the river during the present investigation. The species of *Aeromonas* (*Aeromonas veronii*) was reported, which acts as a fish and opportunistic pathogen to humans [55]. *Vibrio* sp. causes diseases in fish, but being zoonotic also infects birds and animals; consumption of undercooked infected fish causes gastrointestinal problems in humans [10, 18]. *Spiroplasma* has been observed as a severe pathogen of crayfish [99], *Nocardia* is being found in multiple hosts including fish and human [26, 60] and acts as a fish pathogen [3]. *Flavobacterium* sp., *Citrobacter* sp., *Proteus* sp., *Klebsiella* sp., *Comamonas*, *Plesiomonas*, *Ralstonia*, *Chryseobacterium*, *vibrio*, and *Edwardsiella* have been recognized as fish pathogens [97]. *Comamonas*, *Ralstonia* sp., *Ochrobactrum* sp., *Pseudomonas* sp., *Sphingomonas* sp., and *Brevundimonas* sp. are also opportunistic pathogens [72]. *Leclercia adecarboxylata* is also a human opportunistic pathogen affecting immunocompromised hosts [105]. All these pathogenic microbiota were also reported at the sites of Baner rivulet. Furthermore, more deep studies are required to carry on the Baner regarding control of these types of pathogenic microorganisms.

## Conclusion

The first-time metagenomic study of Baner rivulet revealed *Proteobacteria* to be the most dominating phylum at all the sampling sites indicating municipal wastes, human excreta, and anthropogenic pollution. Proportionately, more presence of *Proteobacteria* at S_3_ indicates fecal pollution and more anthropogenic interference at this site. S_3_ is the last point of investigation and falls downstream the river after crossing the main city, so more anthropogenic impact has been observed. As several human, fish, and zoonotic pathogens were found in the water, it is advised to be watchful, periodically disinfect, and use appropriate bio-remedial techniques as the water of Baner has been lifted by the Himachal Pradesh Irrigation and Public Health Department both for drinking and irrigation purposes. However, the Baner also possesses an abundant bacterial profile that holds great promise for developing bioremediation tactics against a variety of harmful substances, such as heavy metals, pesticides, xenobiotic, nitrogen and sulfur, phenol, and toluene metabolism, and also has good potential for probiotics concerned with fish health. The genes of bio-remedial and probiotic bacteria can be identified and exploited through genetic engineering or transformation for healthy aquaculture and fishery sector. As the water is used for drinking and agriculture, it is good that it is being purified of heavy metals and pollutants by natural mechanisms. The findings of the present research may also be used as a basis or case study in the future for supporting further freshwater microbiome research. Additionally, it is possible to recover and clone the expected gene pool for xenobiotic degradation to create new bioremediation techniques in the future.

## Data Availability

The data will be provided on reasonable request.

## Abbreviation

BLAST: Basic local alignment search tool
DNA: Deoxyribonucleic acid
dsDNA: Double-stranded DNA
e-DNA: Environmental DNA
FASTA: Fast-All
KEGG: Kyoto Encyclopedia of Genes and Genomes
MGA: Multiple Genome Aligner
MT: Metallothionein
NCBI: National Centre for Biotechnology Information
NGS: Next-generation sequencing
ORF: Open reading frames
PCR: Polymerase chain reaction
WGS: Whole-genome shotgun sequencing
